# Hospitalization Rates for Phototherapy in Neonates With Transcutaneous Bilirubin (TcB) Values >5.5 mg/dL Below the Phototherapy Threshold

**DOI:** 10.7759/cureus.111810

**Published:** 2026-06-30

**Authors:** Saman Aryal, Olufunto Shonibare, Sheng-Hsin Chen, Laxmi Aryal, Prince O Ansong, Shuja A Shaik, Alexander Rodriguez, Marsha Medows

**Affiliations:** 1 Department of Pediatrics, New York City Health and Hospitals Corporation/Woodhull Medical Center, New York, USA; 2 Department of Neonatology, New York City Health and Hospitals Corporation/Woodhull Medical Center, New York, USA; 3 Department of Pediatrics, New York University Grossman School of Medicine, New York, USA

**Keywords:** bilirubin screening, jaundice neonatal, neonatal hyperbilirubinemia, phototherapy units, transcutaneous bilirubin

## Abstract

Introduction: Neonatal jaundice (hyperbilirubinemia) affects over half of term infants. Although most cases are benign, some progress to severe hyperbilirubinemia, causing acute bilirubin encephalopathy or kernicterus. Early detection is essential. Total serum bilirubin (TSB) is the diagnostic standard, but invasive. Transcutaneous bilirubin (TcB) provides a reliable non-invasive screening tool. The 2022 American Academy of Pediatrics (AAP) guidelines define phototherapy thresholds but do not specify safe discharge bilirubin margins. Understanding outcomes for infants discharged near, but below, these thresholds may help guide follow-up and reduce readmissions.

Objective: To evaluate hospitalization rates for phototherapy among neonates discharged with TcB values >5.5 mg/dL versus ≤5.5 mg/dL below the AAP phototherapy threshold and assess associations with ethnicity and feeding type.

Design/methods: A retrospective cohort study of 1,848 term and near-term infants discharged from a single birth center (September 2022-September 2024) was conducted. The difference between TcB and the AAP phototherapy threshold was calculated, and infants were categorized as >5.5 mg/dL or ≤5.5 mg/dL below the threshold. The primary outcome was rehospitalization for phototherapy within one week. Analyses included chi-square and t-tests.

Results: Among 1,848 infants, 46 (2.5%) were rehospitalized for phototherapy. Rehospitalization occurred in 7.9% of infants discharged ≤5.5 mg/dL below threshold compared with 1.2% of those >5.5 mg/dL below (*χ²*=53.0, p<0.001). Infants discharged closer to the threshold were nearly seven times more likely to be readmitted. Asian and Hispanic infants had higher bilirubin levels and readmission rates (p<0.05). Exclusive breastfeeding showed a trend toward higher rehospitalization rates (3.3%) than formula (1.4%) or mixed feeding (1.6%) (p=0.061).

Conclusion: Infants discharged within 5.5 mg/dL of the phototherapy threshold have a significantly higher risk of rehospitalization for phototherapy. Ethnicity and feeding type may influence risk. Maintaining a discharge margin >5.5 mg/dL and ensuring closer follow-up for higher-risk groups may reduce preventable readmissions and improve management of neonatal jaundice.

## Introduction

Neonatal hyperbilirubinemia is a common condition encountered during the newborn period and remains one of the most frequent causes of post-discharge evaluation and readmission [1]. Although most cases follow a benign course, some infants develop severe hyperbilirubinemia that may progress to acute bilirubin encephalopathy or kernicterus if not recognized and managed appropriately. The updated 2022 American Academy of Pediatrics (AAP) guidelines emphasize universal predischarge bilirubin screening using either transcutaneous bilirubin (TcB) or total serum bilirubin (TSB) measurements, together with risk-based follow-up recommendations. Implementation of these guidelines has been associated with reduced utilization of phototherapy and serum bilirubin testing without a significant increase in outpatient bilirubin monitoring [1].

Several studies have identified factors associated with increased risk of readmission for neonatal hyperbilirubinemia. Predischarge bilirubin level has consistently been shown to be one of the strongest predictors of subsequent significant hyperbilirubinemia [2]. In a prospective cohort study, Prachukthum et al. identified elevated predischarge bilirubin levels, lower gestational age, cesarean delivery, and early postnatal weight loss as important predictors of readmission among term infants with hyperbilirubinemia [3]. Similarly, Xu et al. reported that higher bilirubin levels at the initiation of phototherapy and rebound hyperbilirubinemia were associated with an increased risk of readmission among infants with ABO hemolytic disease [4]. In addition, significant postnatal weight loss has been associated with an increased risk of severe hyperbilirubinemia among readmitted breastfed infants [5]. Collectively, these studies support the importance of predischarge bilirubin assessment in identifying infants at risk for subsequent intervention.

Despite clearly defined thresholds for initiating phototherapy, evidence on clinically meaningful discharge bilirubin thresholds below these thresholds remains limited. Existing studies have largely focused on bilirubin risk zones, absolute bilirubin values, or clinical risk factors rather than specific discharge margins relative to phototherapy thresholds [2-5]. Consequently, uncertainty remains regarding the optimal timing and intensity of follow-up for infants discharged with bilirubin levels below, but near, treatment thresholds.

In our newborn bilirubin follow-up clinic, we observed that many infants discharged with bilirubin levels more than 5.5 mg/dL below the phototherapy threshold required no subsequent intervention, whereas infants discharged closer to the treatment threshold appeared more likely to require additional evaluation or phototherapy. Based on these observations and the absence of a clearly defined discharge safety margin within current guidelines, we selected a threshold of 5.5 mg/dL below the AAP phototherapy threshold for further evaluation as a potentially clinically meaningful marker of post-discharge risk.

Therefore, the primary objective of this study was to compare seven-day re-hospitalization rates for phototherapy between neonates discharged with TcB values ≤5.5 mg/dL and >5.5 mg/dL below the AAP phototherapy threshold. Secondary objectives were to evaluate the associations between demographic and clinical factors, including ethnicity and feeding type, and re-hospitalization risk.

## Materials and methods

Study design and setting

A retrospective cohort study was conducted to evaluate the relationship between predischarge TcB levels and subsequent re-hospitalization for phototherapy. The study population included neonates discharged from Woodhull Medical Center, a community hospital in Brooklyn, New York, between September 2022 and September 2024.

**Figure 1:**
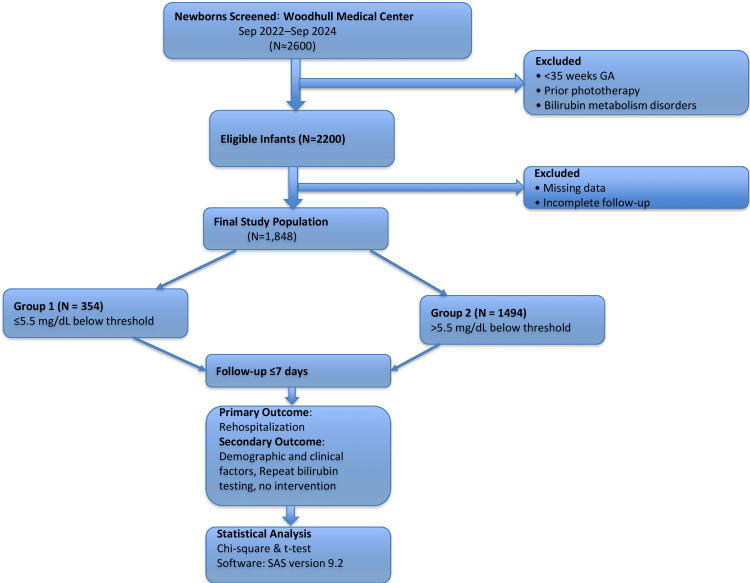
Flowchart illustrating study design, population selection, group allocation, follow-up, outcomes, and statistical analysis.

Approximately 2,600 newborns were screened during the study period. After applying inclusion and exclusion criteria, 1,848 term and near-term infants were included in the final analysis. The process of patient selection, eligibility assessment, group allocation, follow-up, outcome evaluation, and statistical analysis is summarized in Figure 1.

Study population

Eligible participants included term and near-term neonates who underwent predischarge TcB screening. Infants born before 35 weeks' gestation and infants who received phototherapy during the birth hospitalization were excluded. The latter were excluded to specifically evaluate readmission risk among infants initially considered suitable for discharge without phototherapy.

Data collection and variables

Clinical and demographic data were obtained through retrospective review of electronic health records.

The primary independent variable was the threshold-TcB gap, defined as the numerical difference between the age-specific AAP phototherapy threshold and the infant's TcB level at discharge. The AAP phototherapy threshold was determined according to gestational age and clinical risk factors.

Predischarge bilirubin screening was performed using TcB measurements. Confirmatory TSB testing was obtained when clinically indicated according to institutional protocols and AAP recommendations. For study purposes, the threshold-TcB gap was calculated using the pre-discharge TcB value relative to the age-specific AAP phototherapy threshold.

Infants were stratified into two groups based on this threshold-TcB gap: (1) higher-risk discharge group: infants discharged within 5.5 mg/dL or less below the AAP phototherapy threshold (≤5.5 mg/dL below threshold), and (2) lower-risk discharge group: infants discharged more than 5.5 mg/dL below the AAP phototherapy threshold (>5.5 mg/dL below threshold).

Phototherapy thresholds were determined according to the 2022 AAP hyperbilirubinemia guideline using the infant's gestational age, postnatal age at bilirubin measurement, and applicable neurotoxicity risk factors. The threshold-TcB gap was calculated as the difference between the age-specific AAP phototherapy threshold and the pre-discharge TcB value.

The 5.5 mg/dL cutoff was selected because it represents a clinically relevant follow-up stratification point referenced within the AAP hyperbilirubinemia guideline and reflects a practical decision threshold frequently encountered in newborn discharge planning. The 5.5 mg/dL threshold was selected based on observations from our newborn bilirubin follow-up clinic, where infants discharged more than 5.5 mg/dL below the AAP phototherapy threshold frequently required no subsequent intervention, whereas infants discharged closer to the treatment threshold appeared more likely to require additional evaluation or treatment. This threshold was chosen a priori as a clinically meaningful discharge margin for assessing post-discharge risk.

Additional variables included ethnicity, feeding type (exclusive breastfeeding, formula feeding, or mixed feeding), gestational age, and birth weight.

Outcome measures

The primary outcome was re-hospitalization for phototherapy within seven days of discharge. Re-hospitalization for phototherapy was defined as a hospital admission during which phototherapy was initiated according to AAP treatment thresholds.

Secondary outcomes included follow-up visit interventions, repeat bilirubin testing (TcB or TSB), and evaluation of demographic and clinical factors associated with higher bilirubin levels and increased re-hospitalization risk.

Ethnicity was obtained from the electronic medical record and categorized as Hispanic, Asian, Black, White, and Other according to the demographic classification recorded at birth.

Follow-up outcomes were determined through review of electronic medical records and documented outpatient bilirubin follow-up visits within our healthcare system.

Follow-up outcomes were categorized as (1) no intervention required, (2) repeat bilirubin assessment using TcB or TSB, or (3) re-hospitalization for phototherapy within seven days of discharge.

During follow-up, bilirubin assessment was performed in accordance with the 2022 AAP hyperbilirubinemia guideline. Confirmatory TSB measurements were obtained when the TcB value was within 3 mg/dL of the age-specific phototherapy threshold or when the TcB value was ≥15 mg/dL. Infants who did not meet these criteria were managed according to routine clinical assessment and follow-up.

Statistical analysis

Categorical variables, including re-hospitalization status, ethnicity, feeding type, and follow-up outcomes, were compared using chi-square tests. Continuous variables, including gestational age, birth weight, and bilirubin levels, were compared using independent two-sample t-tests.

Analyses were performed using complete-case methodology. Cases with missing values for variables included in a given analysis were excluded from that specific analysis. Missing data were minimal and did not substantially affect the overall cohort size.

Given the limited number of re-hospitalization events, multivariable modeling was not performed. A two-sided p-value of less than 0.05 was considered statistically significant. Statistical analyses were performed using SAS (version 9.2, SAS Institute, Cary, NC).

## Results

A total of 1,848 term and near-term infants met the inclusion criteria and were included in the final analysis. Overall, 46 infants (2.5%) required re-hospitalization for phototherapy within seven days of discharge.

Re-hospitalization by discharge bilirubin margin

Re-hospitalization rates stratified by discharge bilirubin margin are presented in Table 1 and Figure 1. Infants discharged ≤5.5 mg/dL below the AAP phototherapy threshold had significantly higher re-hospitalization rates than those discharged >5.5 mg/dL below the threshold. Re-hospitalization occurred in 28 infants (7.9%) in the ≤5.5 mg/dL group compared with 18 infants (1.2%) in the >5.5 mg/dL group (χ²=53.0, p<0.001). The relative risk of re-hospitalization was 6.56 (95% CI: 3.68-11.74), corresponding to an odds ratio of 7.04 (95% CI: 3.90-12.71).

**Table 1:** Re-hospitalization for phototherapy by discharge bilirubin margin relative to the AAP phototherapy threshold. Infants discharged ≤5.5 mg/dL below the AAP phototherapy threshold had significantly higher rehospitalization rates than infants discharged >5.5 mg/dL below the threshold (χ²=53.0, p<0.001).

Discharge Bilirubin Margin	Re-hospitalized, n (%)	Not Re-hospitalized, n (%)	Total (n)	Chi-square (χ²)	p-value
≤5.5 mg/dL below threshold	28 (7.9)	326 (92.1)	354		
>5.5 mg/dL below threshold	18 (1.2)	1476 (98.8)	1494	53.0	<0.001

Follow-up outcomes

Follow-up outcomes according to discharge bilirubin margin are summarized in Table 2. Infants discharged ≤5.5 mg/dL below the phototherapy threshold were less likely to require no intervention during follow-up than infants discharged >5.5 mg/dL below the threshold (310 infants (87.6%) vs. 1,420 infants (95.0%); p<0.001). Repeat bilirubin assessment using TcB or TSB occurred in 16 infants (4.5%) in the ≤5.5 mg/dL group and 56 infants (3.7%) in the >5.5 mg/dL group. Repeat TcB or TSB measurements during follow-up were obtained according to the 2022 AAP hyperbilirubinemia guideline, with confirmatory TSB testing performed when TcB values were within 3 mg/dL of the phototherapy threshold or ≥15 mg/dL. Re-hospitalization for phototherapy occurred in 28 infants (7.9%) in the ≤5.5 mg/dL group compared with 18 infants (1.2%) in the >5.5 mg/dL group (p<0.001).

**Table 2:** Follow-up outcomes by discharge bilirubin margin. Follow-up outcomes, including no intervention, repeat bilirubin assessment, and rehospitalization for phototherapy, stratified by discharge bilirubin margin.

Follow-up outcome	≤5.5 mg/dL below threshold	>5.5 mg/dL below threshold	Chi-square (χ²)	p-value
No intervention needed	310 (87.6%)	1420 (95.0%)		<0.001
Repeat TcB or TSB	16 (4.5%)	56 (3.7%)		0.5
Rehospitalized for phototherapy	28 (7.9%)	18 (1.2%)	53.0	<0.001

Demographic and clinical factors

Associations between demographic and clinical variables and re-hospitalization risk are summarized in Table 3. Ethnicity was significantly associated with bilirubin levels and re-hospitalization risk. Asian and Hispanic infants demonstrated higher bilirubin levels and higher re-hospitalization rates compared with other ethnic groups (p<0.05).

**Table 3:** Demographic and clinical factors associated with hyperbilirubinemia and rehospitalization. Ethnicity, feeding type, gestational age, and birth weight were evaluated for associations with bilirubin levels and rehospitalization risk.

Variable	Comparison groups	p-value	Key findings
Ethnicity	Asian/Hispanic vs other	<0.05	Higher bilirubin levels and re-hospitalization rates
Feeding type	Breast vs formula vs mixed	0.061	Trend toward higher re-hospitalization
Gestational age	Continuous variable	0.857	No significant association
Birth weight	Continuous variable	0.402	No significant association

Feeding modality showed a trend toward association with re-hospitalization. Exclusively breastfed infants had higher rehospitalization rates than formula-fed or mixed-fed infants (p=0.061). No statistically significant associations were observed between re-hospitalization and gestational age or birth weight.

## Discussion

Neonatal hyperbilirubinemia remains one of the most common causes of post-discharge follow-up and readmission during the newborn period. Although the 2022 AAP guideline provides clear recommendations regarding phototherapy thresholds and follow-up based on pre-discharge bilirubin measurements, the optimal bilirubin margin below the treatment threshold at discharge remains uncertain [6]. In this retrospective cohort of 1,848 term and near-term infants, we found that infants discharged within 5.5 mg/dL of the phototherapy threshold had significantly higher re-hospitalization rates for phototherapy than those discharged at a greater margin. These findings suggest that discharge bilirubin margin may serve as a clinically useful marker for risk stratification after newborn discharge.

Our findings are consistent with previous studies demonstrating that pre-discharge bilirubin levels are among the strongest predictors of subsequent significant hyperbilirubinemia. Bhutani et al. first demonstrated the predictive value of pre-discharge bilirubin screening using an hour-specific bilirubin nomogram, showing that bilirubin levels closer to treatment thresholds are associated with increased risk of later hyperbilirubinemia [2]. Similarly, Newman et al. reported that combining bilirubin measurements with clinical risk factors improves the prediction of subsequent severe jaundice and readmission risk [7]. The markedly higher re-hospitalization rate observed among infants discharged ≤5.5 mg/dL below the phototherapy threshold in our study supports the concept that proximity to treatment thresholds remains an important determinant of post-discharge outcomes even in the era of updated AAP guidelines.

An important aspect of this study is the identification of a practical discharge buffer of 5.5 mg/dL. This threshold was selected based on observed patterns in our newborn bilirubin follow-up clinic, where infants discharged closer to the treatment threshold appeared more likely to require intervention after discharge. While the current AAP guideline recommends follow-up intervals based on the difference between bilirubin levels and phototherapy thresholds, it does not identify a specific margin associated with substantially increased readmission risk [5]. Our findings suggest that a discharge bilirubin margin of ≤5.5 mg/dL may identify a subgroup warranting closer surveillance and earlier follow-up evaluation.

Ethnicity was also associated with bilirubin levels and the risk of rehospitalization. Asian and Hispanic infants demonstrated higher bilirubin levels and increased rates of phototherapy readmission compared with other groups. Similar observations have been reported previously, with genetic and physiologic factors influencing bilirubin metabolism and susceptibility to hyperbilirubinemia [8]. Recognition of these disparities is important because uniform follow-up recommendations may not adequately account for population-specific risk profiles. Incorporating demographic risk factors into post-discharge planning may improve the effectiveness of follow-up strategies and reduce preventable readmissions.

Although feeding type did not reach statistical significance in our cohort, exclusively breastfed infants showed a trend toward higher rehospitalization rates than formula-fed and mixed-fed infants. This finding is consistent with prior literature demonstrating that breastfeeding-associated jaundice may result from delayed establishment of adequate milk intake, increased enterohepatic circulation, and greater postnatal weight loss [4,9]. Salas et al. reported that significant weight loss among breastfed neonates was associated with an increased likelihood of severe hyperbilirubinemia requiring readmission [5]. These findings highlight the importance of lactation support, feeding assessment, and close monitoring of weight trajectories during the early newborn period.

The follow-up outcome analysis revealed that most infants discharged with >5.5 mg/dL below the treatment threshold required no intervention at subsequent follow-up visits. This observation raises the possibility that some lower-risk infants may undergo intensive follow-up with limited clinical benefit. Similar findings have been reported by Petersen et al., who demonstrated that risk-based bilirubin screening strategies can reduce unnecessary healthcare utilization while maintaining patient safety [10]. More recently, implementation studies evaluating the 2022 AAP guideline have reported reductions in phototherapy use and bilirubin testing without evidence of increased adverse outcomes [1]. Together, these findings support the development of more individualized follow-up pathways based on discharge bilirubin margin and other clinical risk factors.

The strengths of this study include a large cohort of newborns managed under a standardized hyperbilirubinemia screening protocol and the use of real-world clinical outcomes. However, several limitations should be acknowledged. First, this was a retrospective single-center study, which may limit generalizability to other populations and practice settings. Second, the observational design precludes the determination of causality. Third, factors such as breastfeeding adequacy, degree of postnatal weight loss, parental health literacy, socioeconomic determinants of healthcare access, transportation barriers, and access to timely outpatient follow-up were not consistently available for analysis and may have influenced outcomes. These variables may contribute to both hyperbilirubinemia severity and healthcare utilization and should be incorporated into future prospective studies. Finally, although the 5.5 mg/dL discharge margin demonstrated strong predictive value in this cohort, external validation in prospective multi-center studies is required before broader implementation.

Follow-up data were obtained by reviewing electronic medical records within our healthcare system. Consequently, re-hospitalizations or follow-up encounters at outside institutions may not have been captured, potentially leading to an underestimation of readmission rates. In addition, because only 46 re-hospitalization events occurred during the study period, multivariable modeling was not performed. Future multi-center studies with larger numbers of outcome events may allow for a more robust assessment of independent predictors of re-hospitalization.

Overall, our findings suggest that discharge bilirubin margin relative to the phototherapy threshold is associated with re-hospitalization risk and may help identify newborns who could benefit from closer post-discharge surveillance. Although the 5.5 mg/dL threshold demonstrated strong predictive value in this cohort, prospective multicenter validation is necessary before it can be incorporated into clinical decision-making or discharge planning protocols.

## Conclusions

In this cohort of term and near-term neonates, discharge bilirubin margin relative to the AAP phototherapy threshold was strongly associated with re-hospitalization for phototherapy. Infants discharged within 5.5 mg/dL of the treatment threshold had a significantly higher risk of readmission compared with those discharged at a greater margin. Additionally, ethnicity and feeding type may further influence this risk.

These findings suggest that discharge bilirubin margin may be a useful component of risk-stratified follow-up strategies. However, the proposed 5.5 mg/dL threshold was derived from a single-center retrospective cohort and should be considered hypothesis-generating. Prospective multicenter studies are needed to validate its reproducibility, generalizability, and clinical utility before widespread adoption as a clinical decision-making tool.
